# Evaluation of Two Multiplexed qPCR Assays for Malaria Detection and Speciation: A Comparative Study With Nested PCR and Microscopy

**DOI:** 10.1155/japr/4950793

**Published:** 2025-02-25

**Authors:** Ahmed A. Muyidi, Musa A. Ayashi, Majed H. Wakid, Maimonah S. Alghanmi, Fadi M. Baakdah, Hattan S. Gattan, Isra M. Alsaady, Muslimah N. Alsulami, Haleema H. Albohiri, Sarah A. Altwaim, Zaki M. Eisa, Thamer M. Brek

**Affiliations:** ^1^Main Laboratory and Blood Bank, Medical and Molecular Microbiology Laboratory, King Faisal Medical City for Southern Region, Abha, Saudi Arabia; ^2^Main Laboratory and Blood Bank, Medical and Molecular Microbiology Laboratory, Prince Mohammad Bin Nasser Hospital, Jizan, Saudi Arabia; ^3^Department of Medical Laboratory Sciences, Faculty of Applied Medical Sciences, King Abdulaziz University, Jeddah, Saudi Arabia; ^4^Special Infectious Agents Unit, King Fahd Medical Research Center, King Abdulaziz University, Jeddah, Saudi Arabia; ^5^Vaccines and Immunotherapy Unit, King Fahd Medical Research Center, King Abdulaziz University, Jeddah, Saudi Arabia; ^6^Department of Biological Sciences, College of Science, University of Jeddah, Jeddah, Saudi Arabia; ^7^Department of Clinical Microbiology and Immunology, Faculty of Medicine, King Abdulaziz University, Jeddah, Saudi Arabia; ^8^Vector-Borne Diseases Laboratories, Public Health Authority, Jazan, Saudi Arabia; ^9^Public Health Laboratory, The Regional Laboratory, and The Central Blood Bank, Jazan Health Directorate, Jazan, Saudi Arabia

**Keywords:** Altona, multiplex PCR, nested PCR, *Plasmodium*, qPCR, Viasure

## Abstract

**Background:** Malaria is a deadly vector-borne parasitic disease spread by the bite of an infective female *Anopheles* mosquito. In routine malaria diagnosis, microscopic examination is generally regarded as the gold standard. Our study sought to evaluate the diagnostic precision of two commercially accessible quantitative PCR (qPCR) kits, in contrast to light microscopy and nested multiplex PCR (NM-PCR).

**Methods:** This cross-sectional study in southwest Saudi Arabia included 92 febrile patients meeting the inclusion criteria. Detection of *Plasmodium* species used light microscopy, NM-PCR, and qPCR kits (RealStar and Viasure). Sensitivity, specificity, positive predictive value (PPV), negative predictive value (NPV), and receiver operating characteristic (ROC) curves were calculated. Statistical analysis was performed using SPSS v25, with significance set at *p* ≤ 0.05.

**Results:** Light microscopy detected 92.4% of cases, NM-PCR detected 73.9%, and RealStar and Viasure detected 92.4% and 95.7%, respectively. Viasure showed the highest sensitivity (97.6%) and NPV (50%), while NM-PCR had superior specificity (71.4%). For species identification, *Plasmodium falciparum* detection was highest with RealStar (85%). Mixed infections were better identified by Viasure (34.6%). RealStar excelled in *Plasmodium vivax* detection (area under the curve [AUC] = 90%). qPCR detected low parasitemia levels missed by microscopy.

**Conclusions:** The qPCR kits, particularly Viasure, demonstrated superior sensitivity for detecting *Plasmodium* species and identifying mixed infections compared to light microscopy and NM-PCR. While light microscopy showed higher specificity and PPV, qPCR effectively detected low parasitemia levels missed by microscopy, highlighting its value in improving malaria diagnostics.

## 1. Introduction

Malaria remains a prominent global health issue, caused by five distinct *Plasmodium* species that can infect humans: *Plasmodium falciparum* (*P. falciparum*), *Plasmodium vivax*, *Plasmodium malariae*, *Plasmodium ovale*, and *Plasmodium knowlesi*. Among these, *P. falciparum* and *P. vivax* are the predominant species, with *P. falciparum* infections being particularly associated with severe malaria complications. In regions with tropical and subtropical climates, malaria remains a substantial cause of morbidity and mortality, particularly in underdeveloped countries [1, 2]. In 2020, 241 million malaria cases were reported worldwide, resulting in 627,000 deaths, mostly in the African region, which accounted for nearly 95% of cases [3]. This high incidence rate poses significant challenges to the World Health Organization's (WHO) global technical strategy for malaria 2016–2030, which is aimed at reducing malaria cases and mortality rates by at least 90% worldwide by 2030 [4]. Saudi Arabia has made remarkable progress in its national malaria control program, successfully reducing annual malaria cases through strategies such as indoor residual spraying, long-lasting insecticide-treated nets, and effective case management [5]. Malaria is still common in the southwestern areas of Jazan and Aseer, even though it has been almost completely wiped out. Malaria spreads randomly in Jazan, and the number of cases has decreased significantly. In 2014, there were only 499 cases reported, but by 2020, that number had risen to 3022 [6, 7]. The primary clinical manifestation of malaria is fever that is a common symptom for most pathogen-related diseases; thus, accurate laboratory testing is essential to prevent misdiagnosis. Microscopy remains the primary diagnostic tool due to its cost-effectiveness, although its accuracy depends on the slide quality and the expertise of the microscopist [8]. Rapid diagnostic tests (RDTs) provided a cost-effective and user-friendly alternative that targets specific *Plasmodium* antigens, but with some limitations such as difficulty detecting light infections and the potential for false positives due to residual antigens [9–15].

Currently, molecular methods like polymerase chain reaction (PCR), including nested multiplex PCR (NM-PCR) and real-time PCR (quantitative PCR [qPCR]), provide significant advantages over traditional techniques [16, 17]. The 18S rRNA gene is commonly targeted for *Plasmodium* species–specific identification, and NM-PCR is recognized for its ability to detect mixed infections and low parasitemia [18]. In Saudi Arabia, NM-PCR has been studied extensively, highlighting its advantages over traditional methods; however, NM-PCR is time-consuming and susceptible to contamination [19–21].

Despite advances in malaria diagnostics, further improvements are necessary, particularly in developing quantitative methods for field studies on asymptomatic malaria. Enhanced understanding of malaria epidemiology is crucial for refining control and elimination strategies [22].

qPCR facilitates real-time detection and quantification of malaria parasites, offering advantages such as rapid processing, enhanced sensitivity and specificity, and the capability to identify multiple *Plasmodium* species in a single assay [23, 24]. To our knowledge, there are no prior studies that have evaluated multiplexed qPCR in detecting mixed *Plasmodium* species infections in the Jazan region of Saudi Arabia. Therefore, this study is aimed at evaluating two commercial multiplexed qPCR assays—RealStar-species assays (Altona Diagnostics, Germany) and Viasure malaria real-time PCR (CerTest Biotec, Spain) for detection and speciation of malaria parasites in EDTA blood samples, using NM-PCR and microscopy as reference methods. Local diagnostic labs could later adopt qPCR as the gold standard method for *Plasmodium* screening if it proves to be less expensive, faster, and more reliable than the other methods.

## 2. Materials and Methods

### 2.1. Ethics Statement

This study was conducted according to the guidelines of the Declaration of Helsinki and approved by the Research and Ethics Committee of the Faculty of Applied Medical Sciences, King Abdulaziz University, Jeddah, Saudi Arabia (FAMS-EC2021-09), and written informed consent was obtained from each participant.

### 2.2. Study Design, Location, and Population

This cross-sectional study was conducted in the Jazan region of southwest Saudi Arabia, involving febrile patients (*n* = 92) seeking medical care at rural and urban medical centers. Participants were selected based on specific inclusion criteria (adults and children with a documented fever or history of fever), and exclusion criteria included those who had received antimalarial treatment within the previous month. Sociodemographic characteristics, including region (urban or rural), gender, nationality (Saudi or non-Saudi), and age group, were recorded.

### 2.3. Blood Film Preparation and Light Microscopy Examination

The malaria light microscopy was carried out at these medical centers in adherence to the protocols recommended by the WHO [25]. Two trained microscopists independently prepared, stained, and examined thick and thin blood films from EDTA blood samples. The thick blood films were examined under a light microscope at 100× magnification to detect malaria parasites. Thin blood films were utilized to quantify parasitemia and accurately identify species, facilitating differentiation between *P. falciparum* and *P. vivax*. The Obare calculator was used to determine whether a third parasitemia count was necessary based on the initial readings.

### 2.4. DNA Extraction and NM-PCR for Malaria

Genomic DNA (gDNA) extraction was performed using the QIAGEN QIAamp 100 DNA Blood Kit (QIAGEN Inc.) according to the manufacturer's instructions. The gDNA was eluted in approximately 100 *μ*L volume of elution buffer, quantified, and assessed for quality using NanoDrop 2000 (Thermo Fisher Scientific, USA) and stored at −20°C. Pan and species-specific NM-PCR analyses were conducted by experienced senior medical technologists who were blinded to the results from light microscopy and qPCR methods. Primers targeting the 18S rRNA gene for *P. falciparum*, *P. vivax*, *P. ovale*, and *P. malariae* were synthesized and used as previously described [18]. Specific primers for *P. knowlesi* were not included. The PCR primer sequences and conditions of NM-PCR were adopted from a previous study (Table 1) [26].

### 2.5. Application of RealStar-Species Assays (Altona) and Malaria Real-Time (Viasure) PCR Kits

RealStar-species assays (Altona) and malaria real-time PCR kits (Viasure) were utilized according to the manufacturers' instructions, with PCR cycling conducted using the ABI PRISM 7500 (Applied Biosystems) for both assays. These assays utilize fluorescence-labeled hydrolysis probes and are designed to detect all five *Plasmodium* species. Samples were considered negative if the fluorescent signal did not exceed the threshold at Cycle 40.

### 2.6. Statistical Analysis

Data entry was done using Microsoft Excel 2016, while data analysis was done using SPSS program Version 25 (IBM Inc., Armonk, NY, USA). Quantitative variables were presented as mean and standard deviation, while qualitative variables were presented as counts and percentages. The one-way ANOVA test was used to compare the means of quantitative variables, while chi-square tests assessed associations between qualitative variables. Sensitivity, specificity, positive predictive value (PPV), and negative predictive value (NPV) were calculated for NM-PCR, light microscopy, and both qPCR kits (RealStar and Viasure), using either NM-PCR or light microscopy as reference methods. Interrater agreement among NM-PCR, light microscopy, and both qPCR kits was assessed using Cohen's kappa statistic, interpreted as follows: ≤ 0 indicates no agreement, 0.01–0.20 suggests none to slight agreement, 0.21–0.40 indicates fair agreement, 0.41–0.60 represents moderate agreement, 0.61–0.80 indicates substantial agreement, and 0.81–1.00 signifies almost perfect agreement. Receiver operating characteristic (ROC) curves and the area under the curve (AUC) were calculated for *P. falciparum* and *P. vivax* using the RealStar and Viasure kits, with light microscopy as the reference. A *p* value of ≤ 0.05 was considered statistically significant. Data visualization was performed using simple and component bar charts, Venn diagrams, and ROC curves as appropriate.

## 3. Results

In this study, we evaluated the performance of two commercial qPCR kits, namely, qPCR RealStar (Altona) and qPCR (Viasure), for screening *Plasmodium* species and detecting mixed infections in febrile patients in the Jazan region, southwest Saudi Arabia, relying on light microscopy and NM-PCR as standards.

### 3.1. Study Population Characteristics

The sociodemographic profile of the febrile patients (*n* = 92) demonstrated a balanced distribution between rural (44.6%) and urban (55.4%) areas. Males constituted the majority (82.6%), with Saudi nationals representing 87.0% of the cohort. Age-wise, 18- to less than 30-year-olds were the most affected age group (48.9%) (Table 2).

### 3.2. Detection of Human *Plasmodium* spp. and Method Performance

The overall performance of each diagnostic method was assessed. Light microscopy identified 85 positive cases (92.4%), while NM-PCR detected 68 cases (73.9%). Both qPCR kits demonstrated comparable performances to light microscopy, with qPCR RealStar (Altona) detecting 85 cases (92.4%) and qPCR (Viasure) identifying 88 cases (95.7%). The prevalence of *P. falciparum* was 52.2% by light microscopy, 51.1% by NM-PCR, 71.7% by qPCR RealStar, and 59.8% by qPCR (Viasure). For *P. vivax*, the prevalence was 12% by light microscopy, 22% by NM-PCR, 9.8% by qPCR RealStar, and 8.7% by qPCR (Viasure). No cases of *P. ovale*, *P. malariae*, or *P. knowlesi* were detected in our study group with any of the four methods used (Table 3).

### 3.3. Diagnostic Accuracy Comparisons

Using light microscopy as a reference, NM-PCR, qPCR RealStar, and qPCR (Viasure) yielded 66, 81, and 83 true positive cases, respectively, alongside true negative cases of 5, 3, and 2. This resulted in sensitivities of 77.6%, 95.3%, and 97.6%, with specificities of 71.4%, 42.9%, and 28.6%. The PPVs were 97.1%, 95.3%, and 94.3%, and NPVs were 20.8%, 42.9%, and 50%, respectively. Cohen's kappa values indicated fair agreement for NM-PCR (0.232), qPCR RealStar (0.382), and qPCR (Viasure) (0.326) (Table 4).

As shown in Table 5, when NM-PCR was used as a reference, light microscopy, qPCR RealStar, and qPCR (Viasure) detected 66, 65, and 66 true positive cases, respectively, with sensitivities of 97.1%, 95.6%, and 97.1% and specificities of 20.8%, 16.7%, and 8.3%. The corresponding kappa values were 0.232, 0.159, and 0.074.

### 3.4. Species Identification Accuracy

**Figure 1:**
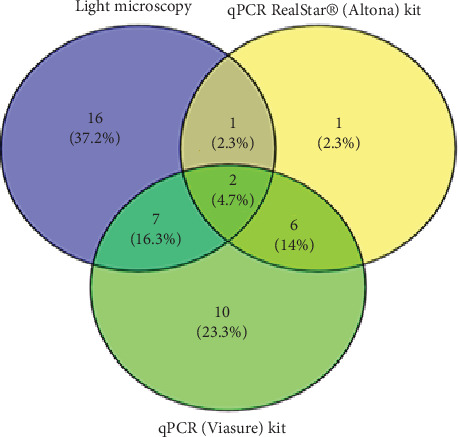
Comparison of results for qPCR RealStar (Altona), qPCR (Viasure), and light microscopy for the diagnosis of mixed malaria.

In assessing species identification for mixed *Plasmodium* spp., true positive cases for NM-PCR, qPCR RealStar, and qPCR (Viasure) were 0, 3, and 9, resulting in sensitivities of 0%, 11.5%, and 34.6%, respectively. The PPVs for these methods were 0%, 30%, and 36%, respectively. For *P. falciparum*, true positive cases were 33, 41, and 36, representing sensitivities of 69%, 85%, and 75%. On the other hand, the true positive cases of *P. vivax* were 11, 4, and 5, resulting in sensitivities of 100%, 36.4%, and 46%, respectively (Table 6 and Figure 1). Notably, as shown in Table 7, when NM-PCR was used as a reference, true positive cases for *P. falciparum* were similar, but no mixed cases were detected.

### 3.5. ROC Curve Analysis

**Figure 2:**
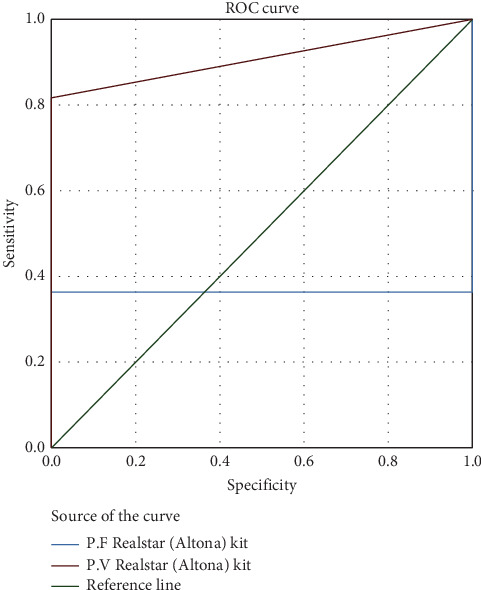
ROC curve for *P. falciparum* RealStar (Altona) and *P. vivax* RealStar (Altona) with light microscopy as a reference method for the diagnosis of malaria cases.

**Figure 3:**
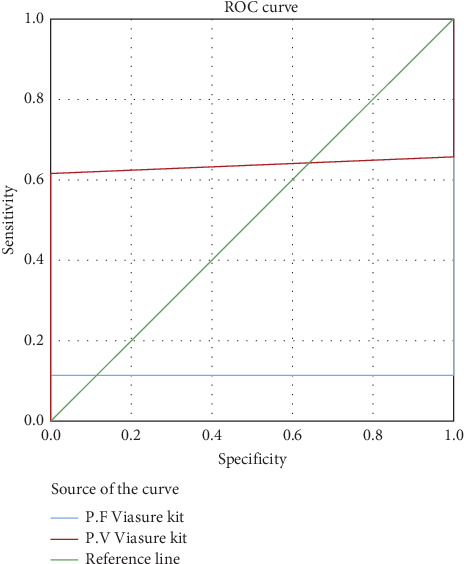
ROC curve for *P. falciparum* (Viasure) and *P. vivax* (Viasure) with light microscopy as a reference method for the diagnosis of malaria cases.

The AUC for the RealStar (Altona) kit was 36.4% for *P. falciparum* and 90.0% for *P. vivax*, indicating excellent accuracy, particularly for *P. vivax* (Figure 2). The AUC for the qPCR (Viasure) kit was 11.5% for *P. falciparum* and 63.5% for *P. vivax*, reflecting moderate accuracy (Figure 3). Overall, the qPCR (Viasure) kit demonstrated higher sensitivity for mixed infections compared to the RealStar (Altona) kit.

### 3.6. Parasitemia Assessment

When evaluating the ability of the qPCR RealStar (Altona) and Viasure kits to assess parasitemia levels in comparison to light microscopy, no significant differences were observed between the performances. The *P. falciparum* RealStar (Altona) kit exhibited the lowest mean value of parasitemia among individuals identified with *P. falciparum* or mixed infections by microscopy. Similarly, the *P. vivax* RealStar (Altona) kit showed the lowest mean value of parasitemia among those diagnosed with *P. vivax* infections by microscopy.

**Figure 4:**
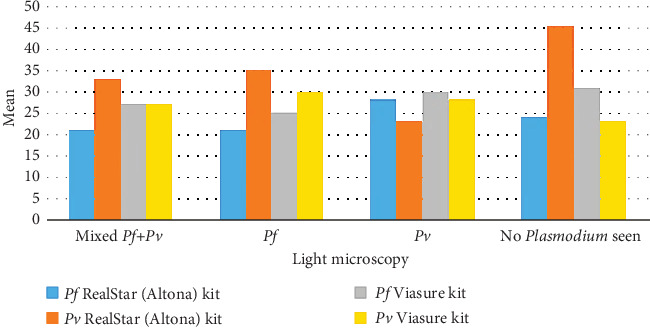
Comparing the qPCR RealStar (Altona) and qPCR (Viasure) in assessing parasitemia levels to light microscopy. *Pf*, *P. falciparum*; *Pv*, *P. vivax*.

The *P. falciparum* Viasure kit demonstrated the lowest mean value among patients with *P. falciparum* infections identified by microscopy. Notably, the *P. vivax* Viasure kit recorded its lowest mean parasitemia values in individuals with no detectable parasites by light microscopy. This highlights qPCR's advantage in detecting low parasite densities, while at higher parasitemia levels, its performance is comparable to light microscopy (Table 8 and Figure 4).

### 3.7. Gender Stratification

When analyzing the data by gender, NM-PCR demonstrated significantly higher specificity and NPV among females (100% and 60%) compared to males (50% and 10.5%) (*p* = 0.015) (Table 9).

## 4. Discussion

Malaria, caused by *Plasmodium* parasites and transmitted through *Anopheles* mosquito bites, is a significant health challenge in regions with high transmission rates, such as southwest Saudi Arabia [27]. Timely and accurate diagnosis is critical for effective disease management. Traditional methods such as light microscopy and NM-PCR have been reported to have limitations in *Plasmodium* sp. screening, highlighting the need for advanced techniques like qPCR, which has been shown to have superior sensitivity and specificity [28].

In our study, we evaluated the diagnostic accuracy of two commercial qPCR kits, qPCR RealStar (Altona) and qPCR (Viasure), compared to light microscopy and NM-PCR among 92 febrile patients. Light microscopy identified 85 positive cases (92.4%), while NM-PCR detected 68 cases (73.9%). Both qPCR kits demonstrated comparable performances, detecting 85 and 88 positive cases, respectively, with positive rates of 92.4% and 95.7%. Notably, qPCR (Viasure) exhibited the highest sensitivity at 97.6%, surpassing both qPCR RealStar (Altona) at 95.3% and light microscopy at 77.6%. These results align with previous findings indicating that molecular methods enhance sensitivity compared to traditional microscopy [29, 30]. Furthermore, when both qPCR kits were compared against light microscopy, they displayed good NPV, indicating their ability to accurately identify individuals without malaria, a critical factor for clinical decision-making and epidemiological studies. Moreover, qPCR (Viasure) had the highest NPV at 50%, followed by the RealStar-species assay at 42.9%. This implies that these qPCR assays are valuable for ruling out malaria cases. However, it is important to highlight that both qPCR RealStar (Altona) and qPCR (Viasure) kits showed low specificities of 42.9% and 28.6%, respectively, when compared to light microscopy.

Given the agreement between both qPCR kits and light microscopy, as indicated by the kappa coefficient, and considering the low specificity of the qPCR kits in our study, it is important to discuss the relevant findings from Ramírez et al., who evaluated the RealStar genus and RealStar species kits against the reference method (NM-PCR) [30]. Their study demonstrated a very high level of concordance, with 99.17% agreement for the RealStar genus kit and 97.5% agreement for the RealStar-species assay. These results suggest that our qPCR kits' specificity may have been compromised when using light microscopy results from real-world settings, potentially due to factors such as varying levels of experience among technicians at the medical centers where the samples were collected.

When compared to NM-PCR, both qPCR kits demonstrated similar or slightly higher sensitivity, with qPCR (Viasure) achieving the highest sensitivity at 97.1%, closely followed by qPCR RealStar (Altona) at 95.6%, which is near the value reported by a previous study [30]. However, it is important to note that both qPCR kits displayed the lowest specificity values when compared using NM-PCR as a reference test (16.7% for qPCR RealStar [Altona] and 8.3% for qPCR [Viasure]).

However, by comparing the overall performance of NM-PCR with that of light microscopy, qPCR RealStar, and qPCR (Viasure), we observed notable differences. NM-PCR detected the lowest number of positive cases (68 cases, 63%) and the highest number of negative cases (24 cases, 22%). In contrast, light microscopy, qPCR RealStar, and qPCR (Viasure) collectively identified approximately 90% of all study cases as positive and less than 8% as negative. This suggests that around 10% of cases identified as negative by NM-PCR may have been false negatives. Notably, when we examined the parasitic index of these cases classified as negative by NM-PCR, most of them had the lowest parasitemia levels detected by both qPCR kits. Consequently, the relatively high rate of false negatives with NM-PCR may have impacted its performance as a reference method, indirectly affecting the specificity of both qPCR kits and light microscopy [31, 32].

Species differentiation and the detection of mixed infections are crucial aspects of malaria diagnosis. Frequently, these infections are overlooked, being wrongly identified as single infections or some species as mixed infections, especially with traditional methods like light microscopy, and this misdiagnosis can still happen when PCR methods are used, particularly if one of the *Plasmodium* species has a low level of parasites in the blood [33, 34]. Failing to correctly identify mixed infections can be problematic, potentially resulting in incorrect treatment, worsening the patient's health, and complicating malaria control efforts in regions where the disease is prevalent. Therefore, the importance of employing accurate molecular methods for detecting these mixed infections cannot be overstated.

In the current study, the prevalence of *P. falciparum*, consistent with the findings of Hawash et al. and Al-Zaydani et al., was 52.2% by light microscopy, 51.1% by NM-PCR, 71.7% by qPCR RealStar (Altona), and 59.8% by qPCR (Viasure) [20, 35]. For *P. vivax*, our results showed a prevalence of 12% by light microscopy, 22.8% by NM-PCR, 9.8% by qPCR RealStar (Altona), and 8.7% by qPCR (Viasure). The performance of these four methods revealed that qPCR RealStar (Altona) exhibited superior accuracy in species differentiation, especially for *P. vivax*, with an accuracy rate of 90.0%. In contrast, qPCR (Viasure) exhibited lower accuracy rates for species differentiation. This emphasizes the importance of selecting the appropriate diagnostic method based on the prevalent *Plasmodium* species in each region for effective malaria control.

The prevalence of mixed *Plasmodium* infections was a notable finding, with 28.3% of cases identified by light microscopy as mixed *P. falciparum* and *P. vivax* infections. However, when assessed by qPCR RealStar (Altona) and qPCR (Viasure), we detected 27% of cases as mixed *P. falciparum* and *P. vivax* infections, although there was some variation between these methods. This high prevalence of mixed infections in our study contrasts with earlier findings by Hawash et al., who employed NM-PCR and reported a much lower proportion (0.7%) of mixed *P. falciparum* and *P. vivax* infections in the same study location [20]. Additionally, a study conducted by Dajem also detected a lower proportion (1.9%) of mixed *P. falciparum* and *P. vivax* infections in the study's site [19]. In contrast, Gupta et al. reported a significantly higher proportion (45.5%) of mixed infections in India [36]. Previous Saudi studies in the Jazan area highlighted that mixed malaria cases have been frequently overlooked [20, 37]. This underscores the importance of utilizing more advanced diagnostic tools, like qPCR, for accurate detection of mixed malaria infections. Furthermore, following previous observations by Hawash et al. [20] and by Dafalla et al. [38], our findings did not detect any cases attributed to either *P. ovale* or *P. malariae*.

We observed some key demographic characteristics among the febrile patients included in our analysis. A significant majority, 82.6%, were males, which may reflect the occupational or lifestyle factors that predispose them to malaria exposure. Furthermore, 87% of the study participants were of Saudi nationality, emphasizing the local context of our research within the Jazan region. Additionally, nearly half of the patients, specifically 48.9%, fell within the age group of 18 to less than 30 years old. This age distribution is notable as it corresponds to a population segment that often engages in outdoor activities and may be at higher risk of malaria transmission. These demographic details provide important context for interpreting our study results and understanding the malaria epidemiology in the region. Our findings align with those reported by other studies conducted in the same region [20, 29, 39]. Additionally, Hawash et al. also observed similar demographic characteristics among febrile patients in their study [20].

The findings demonstrate that while light microscopy and qPCR perform similarly at higher parasitemia levels, qPCR significantly outperforms microscopy in cases of low parasitemia or submicroscopic infections. This is particularly evident with the *P. vivax* Viasure kit, which identified infections in individuals where microscopy failed to detect any parasites. These results align with established thresholds, as microscopy typically detects parasite densities above 50–100 parasites/*μ*L, whereas qPCR can reliably detect much lower densities. This correlation between parasitemia levels and diagnostic method performance is clinically significant. In early or asymptomatic infections, low parasitemia levels are common, and the superior sensitivity of qPCR ensures that such cases are not missed. This is critical for malaria control programs, as undiagnosed low-level infections can contribute to ongoing transmission, particularly in elimination settings.

When we further analyzed the data by stratifying by gender, interesting patterns emerged. When comparing the results for NM-PCR, qPCR RealStar (Altona) kit, and qPCR (Viasure) kit, alongside light microscopy as the gold standard for diagnosing malaria cases, we found that specificity and NPV were notably higher among females (100% and 60%) compared to males (50% and 10.5%) in the NM-PCR subgroup. This difference was statistically significant (*p* = 0.015), highlighting potential gender-related variations in diagnostic accuracy. However, it is important to note that no gender-related effect was observed in the case of the qPCR RealStar (Altona) kit and the qPCR (Viasure) kit when compared to light microscopy, suggesting that the diagnostic performance of these molecular methods may not be influenced by gender.

This study has several strengths. First and foremost, it represents the first comprehensive investigation in the Jazan region of Saudi Arabia that systematically evaluates the performance of two commercial qPCR kits for malaria diagnosis. This region-specific analysis is crucial, given the geographical diversity in malaria prevalence and *Plasmodium* species distribution. Secondly, the study's focus on mixed infections is a noteworthy contribution to the field, as accurately identifying these cases is essential for appropriate treatment and disease control. Furthermore, the inclusion of demographic data provides valuable insights into the local context of malaria transmission, enabling a more comprehensive interpretation of the study's results.

Despite its strengths, this study has certain limitations. One notable limitation is the observed variation in specificity when comparing qPCR results with those of light microscopy. This discrepancy may be attributed to factors such as the experience and skill level of technicians at the medical centers where samples were collected, suggesting a potential source of variability in light microscopy results. Additionally, the study's sample size is relatively small, and further studies with a larger and more diverse population would enhance the generalizability of the findings.

In conclusion, while qPCR offers clear advantages, its high cost and infrastructure requirements limit its feasibility in resource-limited settings, where light microscopy remains more accessible. Selective implementation in reference laboratories or targeted surveillance could enhance diagnostic capacity. This study highlights the potential of qPCR to improve malaria diagnosis and sets the stage for further research initiatives aimed at reducing malaria-related morbidity and mortality, particularly in southwest Saudi Arabia.

## Figures and Tables

**Table 1:** PCR primers specific for the 18S rRNA gene of *Plasmodium* spp. (reproduced from [26]).

**Species**	**Primer**	**Sequence (5**⁣′**-3**⁣′**)**	**Size (bp)**	**PCR program (no. of cycles, denaturation, annealing, and elongation)**
*Plasmodium*	Forward	TTAAAATTGTTGCAGTTAAAACG	1200	25 cycles, 94°C for 1 min, 58°C for 2 min, 72°C for 2 min
	Reverse	CCTGTTGTTGCCTTAAACTTC		
*P. falciparum*	Forward	TTAAACTGGTTTGGGAAAACCAAATATATT	205	30 cycles, 94°C for 1 min, 58°C for 2 min, 72°C for 2 min
	Reverse	ACACAATGAACTCAATCATGACTACCCGTC		
*P. vivax*	Forward	CGCTTCTAGCTTAATCCACATAACTGATAC	120	30 cycles, 94°C for 1 min, 58°C for 2 min, 72°C for 2 min
	Reverse	ACTTCCAAGCCGAAGCAAAGAAAGTCCTTA		
*P. malariae*	Forward	ATAACATAGTTGTACGTTAAGAATAACCGC	144	30 cycles, 94°C for 1 min, 58°C for 2 min, 72°C for 2 min
	Reverse	AAAATTCCCATGCATAAAAAATTATACAAA		
*P. ovale*	Forward	ATCTCTTTTGCTATTTTTTAGTATTGGAGA	800	30 cycles, 94°C for 1 min, 58°C for 2 min, 72°C for 2 min
	Reverse	GGAAAAGGACACATTAATTGTATCCTAGTG		
*P. ovale curtisi* and *P. ovale wallikeri*	Forward	GCTGTAGCTAATACTTGCTTTA	827	25 cycles, 95°C for 1 min, 55°C for 1 min, 72°C for 1 min
	Reverse	TTCACCTCTGACATCTGAATC		

**Table 2:** Sociodemographic characteristics of the patients (*n* = 92).

**Category**	**No. (%)**
Region	
Rural areas	41 (44.6)
Urban areas	51 (55.4)
Gender	
Female	16 (17.4)
Male	76 (82.6)
Nationality	
Saudi	80 (87.0)
Non-Saudi	12 (13.0)
Age (years)	
Less than 18	17 (18.5)
18 to less than 30	45 (48.9)
30 to less than 40	19 (20.7)
40 or over	11 (12.0)

**Table 3:** Overall performance of light microscopy, NM-PCR, qPCR RealStar (Altona), and qPCR (Viasure) in the detection of malaria cases (*n* = 92).

**Method**	**Positive no. (%)**				**Negative no. (%)**
	**Total**	***Pf***	***Pv***	**Mixed**	**Total**
Light microscopy	85 (92.4)	48 (52.2)	11 (12.0)	26 (28.3)	7 (7.6)
NM-PCR	68 (73.9)	47 (51.1)	21 (22.8)	0 (0)	24 (22.0)
qPCR RealStar (Altona)	85 (92.4)	66 (71.7)	9 (9.8)	10 (10.9)	7 (7.6)
qPCR (Viasure)	88 (95.7)	55 (59.8)	8 (8.7)	25 (27.2)	4 (4.3)

Abbreviations: *Pf*, *P. falciparum*; *Pv*, *P. vivax*.

**Table 4:** Comparison of results for NM-PCR, qPCR RealStar (Altona), and qPCR (Viasure) using light microscopy as a reference for the detection of malaria cases (*n* = 92).

**Method**	**TP (no.)**	**TN (no.)**	**Sensitivity (%)**	**Specificity (%)**	**PPV (%)**	**NPV (%)**	**Kappa**
NM-PCR	66	5	77.6	71.4	97.1	20.8	(0.232) fair agreement
qPCR RealStar (Altona)	81	3	95.3	42.9	95.3	42.9	(0.382) fair agreement
qPCR (Viasure)	83	2	97.6	28.6	94.3	50.0	(0.326) fair agreement

Abbreviations: NPV, negative predictive value; PPV, positive predictive value; TN, true negative; TP, true positive.

**Table 5:** Comparison of results for light microscopy, qPCR RealStar, and qPCR (Viasure) using NM-PCR as a reference for the detection of malaria cases (*n* = 92).

**Method**	**TP (no.)**	**TN (no.)**	**Sensitivity (%)**	**Specificity (%)**	**PPV (%)**	**NPV (%)**	**Kappa**
Light microscopy	66	5	97.1	20.8	77.6	71.1	(0.232) fair agreement
qPCR RealStar (Altona)	65	4	95.6	16.7	76.5	57.1	(0.159) no agreement
qPCR (Viasure)	66	2	97.1	8.3	75.0	50.0	(0.074) no agreement

Abbreviations: NPV, negative predictive value; PPV, positive predictive value; TN, true negative; TP, true positive.

**Table 6:** Comparison of results for NM-PCR, qPCR RealStar (Altona), qPCR (Viasure), and light microscopy as the reference test for speciation and identification of mixed malaria cases (*n* = 92).

**Method**	**Light microscopy**									
	**Mixed (*Pf + Pv*)**		***Pf***		***Pv***		**No parasite seen**		**Total**	
	**No.**	**Sensitivity (%)**	**No.**	**Sensitivity (%)**	**No.**	**Sensitivity (%)**	**No.**	**Sensitivity (%)**	**No.**	**%**
NM-PCR										
*Pf*	12	46.2	33	68.8	0	0	2	28.6	47	51.1
*Pv*	10	38.5	0	0	11	100	0	0	21	22.8
Negative	4	15.4	15	31.3	0	0	5	71.4	24	26.1
qPCR RealStar (Altona)										
Mixed (*Pf + Pv*)	3	11.5	5	10.4	2	18.2	0	0	10	10.9
*Pf*	17	65.4	41	85.4	4	36.4	4	57.1	66	71.7
*Pv*	5	19.2	0	0	4	36.4	0	0	9	9.8
Negative	1	3.8	2	4.2	1	9.1	3	42.9	7	7.6
qPCR (Viasure)										
Mixed (*Pf + Pv*)	9	34.6	12	25.0	3	27.3	1	14.3	25	27.2
*Pf*	14	53.8	36	75.0	2	18.2	3	42.9	55	59.8
*Pv*	2	7.7	0	0	5	45.5	1	14.3	8	8.7
Negative	1	3.8	0	0	1	9.1	2	28.6	4	4.3
Total	26	100	48	100	11	100	7	100	92	100

Abbreviations: *Pf*, *P. falciparum*; *Pv*, *P. vivax*.

**Table 7:** Comparison of results for light microscopy, qPCR RealStar (Altona), qPCR (Viasure), and NM-PCR as the reference method for speciation of malaria cases (*n* = 92).

**Method**	**NM-PCR**							
	***Pf***		***Pv***		**Negative**		**Total**	
	**No.**	**Sensitivity (%)**	**No.**	**Sensitivity (%)**	**No.**	**Sensitivity (%)**	**No.**	**%**
Light microscopy								
Mixed (*Pf* + *Pv*)	12	25.5	10	47.6	4	16.7	26	28.3
*Pf*	33	70.2	0	0	15	62.5	48	52.2
*Pv*	0	0	11	52.4	0	0	11	12.0
No parasite seen	2	4.3	0	0	5	20.8	7	7.6
qPCR RealStar (Altona)								
Mixed (*Pf* + *Pv*)	5	10.6	4	19.0	1	4.2	10	10.9
*Pf*	41	87.2	6	28.6	19	79.2	66	71.7
*Pv*	0	0	9	42.9	0	0	9	9.8
Negative	1	2.1	2	9.5	4	16.7	7	7.6
qPCR (Viasure)								
Mixed (*Pf* + *Pv*)	12	25.5	9	42.9	4	16.7	25	27.2
*Pf*	35	74.5	3	14.3	17	70.8	55	59.8
*Pv*	0	0	7	33.3	1	4.2	8	8.7
Negative	0	0	2	9.5	2	8.3	4	4.3
Total	47	100	21	100	24	100	92	100

Abbreviations: *Pf*, *P. falciparum*; *Pv*, *P. vivax*.

**Table 8:** The ability of the qPCR RealStar (Altona) and qPCR (Viasure) to evaluate the level of parasitemia as compared to light microscopy.

**Method**	**Light microscopy**							
	**Mixed (*Pf* + *Pv*)**		***Pf***		***Pv***		**No parasite seen**	
	**Mean**	**SD**	**Mean**	**SD**	**Mean**	**SD**	**Mean**	**SD**
*Pf* RealStar (Altona)	21_a_	8	21_a_	6	28_a_	9	24_a_	12
*Pv* RealStar (Altona)	33_a_	10	35_a_	7	23_a_	4	45	—
*Pf* (Viasure)	27_a_	8	25_a_	5	30_a_	7	31_a_	11
*Pv* (Viasure)	27_a_	9	30_a_	10	28_a_	4	23_a_	16

*Note:* Values in the same row not sharing the same subscript are significantly different at *p* < 0.05 for all pairwise comparisons within a row using the Bonferroni correction.

Abbreviations: *Pf*, *P. falciparum*; *Pv*, *P. vivax*; SD, standard deviation.

**Table 9:** Comparison of results for NM-PCR, qPCR RealStar (Altona), qPCR (Viasure), and light microscopy as a gold standard for diagnosis of malaria cases after stratification regarding gender.

**Method**	**Light microscopy**					
	**Positive no.**	**Negative no.**	**Sensitivity (%)**	**Specificity (%)**	**PPV (%)**	**NPV (%)**
NM-PCR						
Positive						
Female	11	0	84.6	100⁣^∗^	100	60.0⁣^∗^
Male	55	2	76.4	50.0⁣^∗^	96.5	10.5⁣^∗^
Negative						
Female	2	3				
Male	17	2				
qPCR RealStar (Altona)						
Positive						
Female	12	1	92.3	66.7	92.3	66.7
Male	69	3	95.8	25.0	95.8	25.0
Negative						
Female	1	2				
Male	3	1				
qPCR (Viasure)						
Positive						
Female	13	2	100	33.3	86.7	100
Male	70	3	97.2	25.0	95.9	33.3
Negative						
Female	0	1				
Male	2	1				

Abbreviations: NPV, negative predictive value; PPV, positive predictive value.

⁣^∗^Significant at *p* < 0.05 using the chi-square test.

## Data Availability

The data that support the findings of this study are available from the corresponding author upon reasonable request.
